# High prevalence of SARS-CoV-2 antibodies in pregnant women after the second wave of infections in the inner-city of Johannesburg, Gauteng Province, South Africa

**DOI:** 10.1016/j.ijid.2022.10.036

**Published:** 2022-12

**Authors:** Shobna Sawry, Jean Le Roux, Nicole Wolter, Philile Mbatha, Jinal Bhiman, Jennifer Balkus, Anne von Gottberg, Cheryl Cohen, Matthew Chersich, Malolo Kekana, Thatcher Ndlovu, Angela Shipalana, Wendy Mthimunye, Faeezah Patel, Hermien Gous, Sibongile Walaza, Stefano Tempia, Helen Rees, Lee Fairlie

**Affiliations:** 1 Wits Reproductive Health and HIV Institute, Faculty of Health Sciences, University of the Witwatersrand, Johannesburg, South Africa; 2 Centre for Respiratory Diseases and Meningitis, National Institute for Communicable Diseases, a division of the National Health Laboratory Service, Johannesburg, South Africa; 3 School of Pathology, Faculty of Health Sciences, University of the Witwatersrand, Johannesburg, South Africa; 4 Department of Epidemiology, University of Washington School of Public Health, Seattle, United States of America; 5 Department of Pathology, Faculty of Health Sciences, University of Cape Town, Cape Town, South Africa; 6 School of Public Health, Faculty of Health Sciences, University of the Witwatersrand, Johannesburg, South Africa

**Keywords:** SARS-CoV-2 seroprevalence, Serosurveys, HIV, South Africa, COVID-19, Pregnant women

## Abstract

**Objectives:**

After South Africa's second wave of COVID-19, this study estimated the SARS-CoV-2 seroprevalence among pregnant women in inner-city Johannesburg, South Africa.

**Methods:**

In this cross-sectional survey, 500 pregnant women who were non-COVID-19-vaccinated (aged ≥12 years) were enrolled, and demographic and clinical data were collected. Serum samples were tested using the Wantai SARS-CoV-2 spike antibody enzyme-linked immunosorbent assay and Roche Elecsys® anti-SARS-CoV-2 nucleocapsid antibody assays. Seropositivity was defined as SARS-CoV-2 antibodies on either (primary) or both (secondary) assays. Univariate Poisson regression assessed risk factors associated with seropositivity.

**Results:**

The median age was 27.4 years, and HIV prevalence was 26.7%. SARS-CoV-2 seroprevalence was 64.0% (95% confidence interval [CI]: 59.6-68.2%) on the primary and 54% (95% CI: 49.5-58.4%) on the secondary measure. Most (96.6%) women who were SARS-CoV-2-seropositive reported no symptoms. On the Roche assay, we detected lower seroprevalence among women living with HIV than women without HIV (48.9% vs 61.7%, *P*-value = 0.018), and especially low levels among women living with HIV with a clusters of differentiation 4 <350 cells/ml compared with women without immune suppression (22.2% vs 56.4%, prevalence rate ratio = 0.4; 95% CI: 0.2-0.9; *P*-value = 0.046).

**Conclusion:**

Pregnant women attending routine antenatal care had a high SARS-CoV-2 seroprevalence after the second wave in South Africa, and most had asymptomatic infections. Seroprevalence surveys in pregnant women present a feasible method of monitoring the course of the pandemic over time.

## Highlights

• A SARS-CoV-2 seroprevalence study was conducted after the second wave of COVID infections.
• The study included pregnant women in two urban antenatal clinics in inner-city Johannesburg.
• A total of 64% of pregnant women were SARS-CoV-2 seropositive; most (96.6%) were asymptomatic.
• No association between HIV status and SARS-CoV-2 seropositivity was found.
• Only 60% of pregnant women reported being willing to vaccinate.

## Introduction

In South Africa, by the end of the second COVID-19 wave in early February 2021, which driven largely by the Beta variant (501Y.V2), almost 1.5 million COVID-19 cases and 47,000 deaths were recorded (National Institute for Communicable Diseases [NICD], 2021). By the end of September 2021, as South Africa exited the third wave, which was dominated by the Delta variant, just over 2.9 million diagnosed cases and more than 87,000 COVID-19-related deaths were reported (NDoH, 2021). Of these, more than one-third of cases and a quarter of deaths were from the Gauteng Province, the most densely populated province in South Africa, with the City of Johannesburg accounting for almost one-third of diagnosed cases (NDoH, 2021, NICD, 2021). However, there remain major gaps in knowledge about the epidemiological parameters of SARS-CoV-2 in South Africa, and case ascertainment through reverse transcription-polymerase chain reaction (RT-PCR)-based case identification may substantially underestimate the extent of infections. Once-off and serial seroprevalence studies may address this gap because they may be a more accurate indicator of population-level attack rate than diagnostic testing and could contribute to more accurate estimates of population immunity acquired by natural infection or through vaccination.

Sentinel surveillance of infectious diseases among pregnant women has previously been used as an indicator of disease burden at a population level (Fairlie *et al.*, 2020). Repeated serosurveys among pregnant women are used extensively to track the HIV epidemic in sub-Saharan Africa and elsewhere and to derive population-based HIV prevalence estimates (Eaton *et al.*, 2014; GBD 2016 Mortality Collaborators, 2017; Sangal *et al.*, 2018). Sentinel serosurveillance among pregnant women could potentially serve as a proxy for the SARS-CoV-2 disease burden in the broader community (Fairlie *et al.*, 2020). Findings could be characterized by HIV status, age, and other potential risk factors for infection. Surveys of pregnant women offer advantages over other methods, such as household surveys, which are logistically challenging, costly, often have high refusal rates, may have a low yield, and are unable to give local estimates unless sample sizes are considerable (Gutreuter *et al.*, 2019; Kirakoya-Samadoulougou *et al.*, 2016; Marsh *et al.*, 2011). Between the second and third waves of the COVID-19 pandemic in South Africa, this study aimed to estimate the SARS-CoV-2 seroprevalence among pregnant women in urban antenatal clinics in inner-city Johannesburg, Gauteng, by conducting a cross-sectional serological survey. As a secondary objective, we also evaluated the performance of the Wantai SARS-CoV-2 antibody enzyme-linked immunosorbent assay (ELISA) compared with the Roche Elecsys® anti-SARS-CoV-2 assay for antibody detection.

## Methods

### Study setting

This cross-sectional seroprevalence survey was conducted in Hillbrow, a densely populated inner-city neighborhood of Johannesburg characterized by overcrowded high-rise buildings, large numbers of both documented and undocumented migrants, high levels of unemployment, high prevalence of tuberculosis, and diseases linked to poor water quality and sanitation (Frith, 2011; Rees *et al.*, 2017). The HIV prevalence among pregnant women is estimated at just over 30% in the City of Johannesburg (Woldesenbet *et al*., 2020). Two antenatal care facilities based in the inner-city of Johannesburg were included in the study: Shandukani Midwife Obstetric Unit and Esselen Street Clinic. These clinics are about 500 meters apart and provide antenatal services to the same community, majority of whom reside in the Hillbrow area.

### Participants

Pregnant women attending antenatal care aged ≥12 years were eligible for inclusion in the study if they had no acute illness and had not previously participated in a COVID-19 vaccine trial. The field staff approached women individually in the order that they arrived at the clinic to assess each woman's willingness and eligibility to participate in the study. Women who refused participation were replaced by the next woman who came to the clinic. Willing and eligible women completed written informed consent procedures and were enrolled over a 12-week period from March 17 to June 9, 2021, until the target sample size was achieved.

### Data sources

Participants completed a brief interviewer-administered questionnaire covering basic demographic and health characteristics, a COVID-19 symptom screen, and a history of SARS-CoV-2 infection. Any suspected cases of COVID-19 were referred for SARS-CoV-2 testing, according to Department of Health guidelines. Participants were also asked whether they had received a vaccine for COVID-19 and to provide details of such vaccination. In South Africa, only health care workers were offered vaccinations from February 17, 2021. Subsequently, vaccinations were introduced to the general population using an age-based phased approach, starting with persons over the age of 60 years on May 17, 2021.

Data on HIV and syphilis status, parity, gravidity, gestational age, and suburb of residence, collected as part of standard of care, were extracted directly from the patient-held antenatal clinic card. HIV viral loads and clusters of differentiation (CD4+) T cell counts for women living with HIV (WLHIV) were extracted from the National Health Laboratory Service LabTrak platform. A viral load of ≥100 copies/ml was categorized as elevated, whereas a CD4+ T cell count of <350 counts/ml was categorized as immunosuppressed.

Serum samples were collected and sent to the NICD for testing. Samples were tested in parallel using two different ELISAs to increase the positive predictive value, given the expected prevalence of this virus at the time of conducting the study (Amanat *et al.*, 2020), namely, the Wantai SARS-CoV-2 Ab ELISA (Beijing Wantai Biological Pharmacy Enterprise Co. Ltd, Beijing, China) and the Roche Elecsys® anti-SARS-CoV-2 (Roche Diagnostics, Rotkreuz, Switzerland) serology test kit. The former measures total antibodies (immunoglobulin [Ig]M, IgG, and IgA) against the receptor binding domain in the S1 subunit of the spike protein (anti-S) (FDA, 2020). The latter, however, detects total antibodies (IgM, IgG, and IgA) against SARS-CoV-2 using a recombinant nucleocapsid (N) protein (anti-N) (Roche, 2020).

### Sample size

The sample size was calculated for a 95% confidence interval (CI) and an estimated 30% SARS-CoV-2 seropositivity rate. The 95% CI for a sample of 500 women under these assumptions was 26-34%.

### Statistical analysis

Descriptive statistics were used to characterize the study population. For continuous data with a non-normal distribution, medians and interquartile ranges (IQRs) were computed. For categorical data, proportions with 95% CIs were computed, and the differences between groups were evaluated using either a chi-squared test or prevalence rate ratios with associated 95% CI. SARS-CoV-2 seropositivity was determined in two ways: (i) detection of SARS-CoV-2 antibodies on *either* Wantai or Roche Elecsys assay (primary measure), or (ii) detection of SARS-CoV-2 antibodies on both Wantai and Roche Elecsys assays (secondary measure). The proportion of women with SARS-CoV-2 antibody detection (as described previously) and associated 95% CI were computed, stratified by HIV and immune status. As a secondary objective of this study, the Roche Elecsys assay was used as the reference to calculate the sensitivity, specificity, positive predictive value, and negative predictive value with associated 95% CI for the Wantai assay. Although both assays have previously shown good performance regarding sensitivity, the Roche Elecsys anti-N assay showed a higher specificity and intraclass correlation coefficient, and thereby, less variability than the Wantai assay in an evaluation of commercially available high-throughput SARS-CoV-2 serologic assays (Stone *et al.*, 2022). In addition, the Roche Elecsys assay has shown better performance in another South African study, where the Roche assay did not detect antibodies in 3% of samples tested, whereas the Wantai assay did not detect antibodies in 9% (Wolter *et al.*, 2022).

A Cohen κ for inter-rater agreement between the two assays was computed (Byrt, 1996). The epidemic curve was derived from weekly incident COVID-19 cases reported to the NICD for the City of Johannesburg's Region F, where the two study sites are situated.

The univariate Poisson regression, with robust standard errors, was used to assess the association of demographic and clinical factors with SARS-CoV-2 seropositivity. Results are presented as prevalence rate ratios (PRRs) through exponentiation of coefficients with 95% CI. The same methods were used to assess factors associated with willingness to be vaccinated. For multivariable analyses, independent variables with a *P*-value ≤0.25 in univariate analyses were considered for inclusion in the model (Hosmer and Lemeshow, 2000). All data were captured directly onto the REDCap (Research Electronic Data Capture) data platform (Harris *et al.*, 2009), hosted at the University of the Witwatersrand in South Africa. Analyses were performed using Stata Version 15.1 (Stata Corp, College Station, TX, USA).

## Results

### Participant characteristics and SARS-CoV-2 history

**Figure 1:**
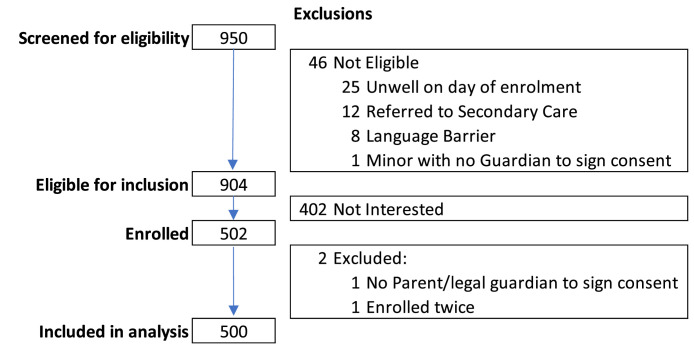
Flow diagram of the number of pregnant women screened, eligible and included in the study.

During the study period, 950 pregnant women were screened, 904 were eligible, 402 declined participation, 502 (55.6%) were enrolled, and two women were subsequently excluded from the study; no parent or legal guardian was available to sign the consent form for a minor's participation and a duplicate enrollment (Figure 1). The median age of the participants was 27.4 years (IQR: 23.6-32.1). The study sample comprised 50.6% South Africans and 46.2% Zimbabwean foreign nationals. More than half (52.4%) of the women were in their third trimester of pregnancy, with a median gestational age of 27 weeks (IQR: 21-34) at the time of enrollment. The prevalence of HIV among women enrolled was 26.7 (Table 1).

**Table 1:** Demographic and clinical characteristics of participants at the time of enrollment, March-June 2021.

Characteristics	Number (proportion, %N) N = 500
Month of enrollment	
March 2021	110 (22.0)
April 2021	170 (34.0)
May 2021	177 (35.4)
June 2021	43 (8.6)
Median age (IQR), in years	27.37 (23.62-32.06)
Age group, in years	
15-19	27 (5.4)
20-24	148 (29.6)
25-29	150 (30.0)
30-34	106 (21.2)
35-39	58 (11.6)
≥40	11 (2.2)
Nationality	
South African	253 (50.6)
Zimbabwean	231 (46.2)
Other	16 (3.2)
Parity	
0	182 (36.8)
1	176 (35.6)
2-4	135 (27.3)
≥ 5	1 (0.2)
Gravidity	
1	156 (31.6)
2-4	320 (64.8)
≥ 5	18 (3.6)
Median gestational age (IQR), in weeks	27 (21-34)
Trimester of pregnancy	N = 494
First trimester	12 (2.4)
Second trimester	223 (45.1)
Third trimester	259 (52.4)
HIV status	N = 491
Positive	131 (26.7)
Negative	360 (73.3)
HIV viral load (copies/ml)	N = 81
<100	69 (85.2)
≥100	12 (14.8)
CD4+ T cells (count/ml)	N = 57
≥350	39 (68.4)
<350	18 (31.6)
Syphilis status	N = 461
Positive	17 (3.7)
Negative	444 (96.3)
Prior positive SARS-CoV-2 test	
Yes	6 (1.2)
No	494 (98.8)
Symptoms suggestive of any previous COVID infection (since Mar 2020)	
Yes	19 (3.8)
No	481 (96.2)
Willing to take COVID-19 vaccine when available	
Yes	296 (59.4)
No/Unsure	202 (40.6)

IQR, interquartile range

Since March 2020, 19 (3.8%) women reported symptoms suggestive of SARS-CoV-2 infection. Of these women, 11 (57.9%) were tested for SARS-CoV-2 at the time of those symptoms, and two (18.2%) reported that they tested RT-PCR-positive. A further 50 women who did not report any COVID-19-related symptoms previously had a SARS-CoV-2 diagnostic test, four (8.0%) of whom tested SARS-CoV-2 RT-PCR-positive. No participants had been vaccinated before study participation.

### Seroprevalence of SARS-CoV-2

SARS-CoV-2 seroprevalence on the primary measure using detection on either the Wantai or the Roche Elecsys assay was 64.0% (95% CI: 59.6-68.2%), whereas the seroprevalence on the secondary measure using detection of antibodies on both the Wantai and the Roche Elecsys assay was 54% (95% CI: 49.5-58.4%) (Table 2). The prevalence of SARS-CoV-2 antibodies among pregnant women was similar for the Wantai (59.6%; 95% CI: 55.2-63.9%) and Roche Elecsys (58.4%; 95% CI: 53.9-64.0%) assays, independently (Table 2). However, of the 208 women who were seronegative on the Roche Elecsys assay, 28 (13.5%) were seropositive on the Wantai assay; whereas of the 292 women who were seropositive on the Roche Elecsys assay, 22 (7.5%) were seronegative on the Wantai assay. The overall inter-rater agreement between the two tests was 90% with a Cohen κ = 0.79; indicating good inter-rater agreement (Byrt, 1996). Using the Roche Elecsys assay as the reference test, the Wantai assay's performance was high, with a sensitivity of 92.5% (95% CI: 88.8-95.2%), specificity of 86.5% (95% CI: 81.1-90.9%), positive predictive value of 90.6% (95% CI: 86.7-93.7%), and negative predictive value of 89.1% (95% CI: 84.0-93.0%) (Table 3). Among WLHIV compared with women without HIV, the sensitivity and negative predictive values were higher, whereas the specificity and positive predictive values were lower (Table 3).

**Table 2:** Prevalence of SARS-CoV-2 antibodies by test type and by primary and secondary seroprevalence measures, among pregnant women in Johannesburg, South Africa, March-June 2021.

		SARS-CoV-2 Seropositivity			
	N	Wantai assay n (%N)	Roche assay n (%N)	Primary: EITHER Wantai or Roche Assay n (%N)	Secondary: BOTH Wantai AND Roche Assay n (%N)
Overall	500	298 (59.6)	292 (58.4)	320 (64.0)	270 (54.0)
95% confidence interval		55.2-63.9	53.9-62.8	59.6-68.2	49.5-58.4
HIV status					
Positive	131	73 (55.7)	64 (48.9)	76 (58.0)	61 (46.6)
Negative	360	220 (61.1)	222 (61.7)	238 (66.1)	204 (56.7)
Unknown	4	4 (100.0)	4 (100.0)	4 (100.0)	4 (100.0)
*P*-value		0.298	0.018	0.117	0.178
HIV viral load (copies/ml)^a^					
<100	69	35 (50.7)	33 (47.8)	36 (52.2)	32 (46.4)
≥100	12	6 (50.0)	6 (50.0)	7 (58.3)	5 (41.7)
*P*-value		0.963	0.888	0.681	0.824
CD4+ T cells (count/ml)^a^					
≥350	39	23 (58.9)	22 (56.4)	24 (61.5)	21 (53.9)
<350	18	7 (38.9)	4 (22.2)	7 (38.9)	4 (22.2)
*P*-value		0.203	0.046	0.157	0.105

a Of the 131 women living with HIV, viral load results were only available for 81 (62%) participants and CD4 results were available for 57 (44%) participants, up to 3 months prior to study participation. CD, clusters of differentiation

**Table 3:** Performance of Wantai SARS-CoV-2 antibody ELISA test compared to the Roche Elecsys anti-SARS-CoV-2 ELISA test as the gold standard method.

	All women N = 500	HIV-positive N = 131	HIV-negative N = 360
Wantai +/ Roche + Sensitivity (95% CI)	270/292 92.5 (88.8-95.2)	61/64 95.3 (86.9-99.0)	204/222 91.9 (87.5-95.1)
Wantai -/Roche- Specificity (95% CI)	180/208 86.5 (81.1-90.9)	55/67 82.1 (70.8-90.4)	122/138 88.4 (81.9-93.2)
Roche+/Wantai+ Positive predictive value (95% CI)	270/298 90.6 (86.7-93.7)	61/73 83.6 (73-91.2)	204/220 92.7 (88.5-95.8)
Roche-/Wantai- Negative predictive value (95% CI)	180/202 89.1 (84.0-93.0)	55/58 94.8 (85.6-98.9)	122/140 87.1 (80.4-92.2)

CI, confidence interval; ELISA, enzyme-linked immunosorbent assay

Of the 496 women with a known HIV status, 131 were living with HIV. Of these, 76 (58.0%) were seropositive using the primary measure, and 61 (46.6%) using the secondary measure of seropositivity. Women without HIV were significantly more likely to be seropositive on the Roche Elecsys assay than WLHIV (61.7% and 48.9%, respectively; *P*-value = 0.018). On the Wantai assay, 55.7% of those without HIV and 61.1% of WLHIV were seropositive (*P*-value = 0.298). For the 57 WLHIV who had available CD4+ cell counts, those with a low count (<350 count/ml) were significantly less likely to be seropositive on the Roche Elecsys assay (PRR = 0.4; 95% CI: 0.2-0.9, *P*-value = 0.046) than women with higher CD4+ counts. Of the 18 WLHIV with low CD4+ cell counts, four were seropositive on both assays, whereas an additional three were positive on the Wantai assay (Table 2). All six women who were SARS-CoV-2-positive on a previous RT-PCR test were also seropositive, with the time since SARS-CoV-2 RT-PCR positivity ranging from 3 to 13 months (Table 2).

**Figure 2:**
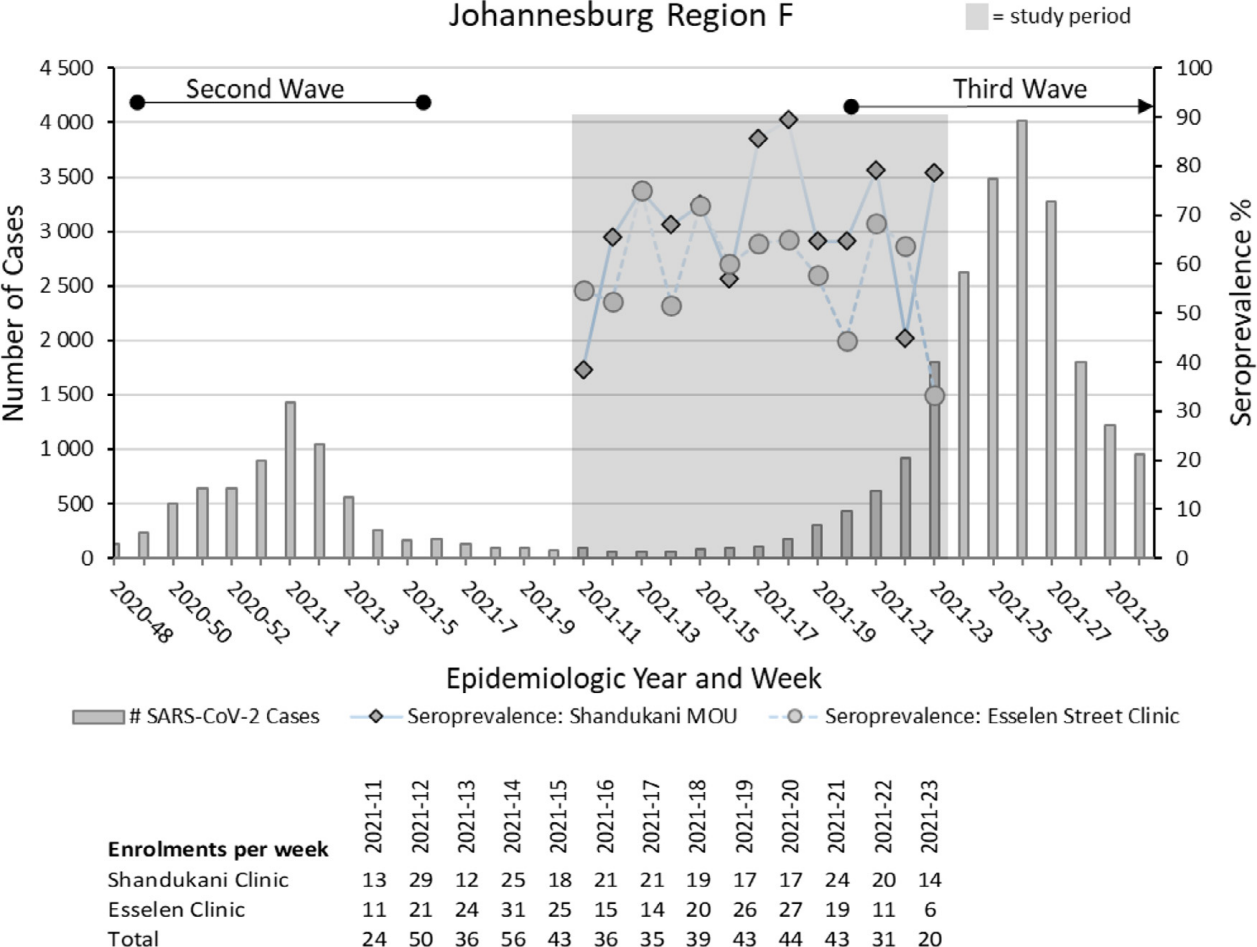
Proportion of SARS-CoV-2 seropositive pregnant women per epidemiologic week at Shandukani Midwife Obstetric Unit and the Esselen Street Clinic over the study period (17 March 2021 to 9 June 2021), plotted against the backdrop of the SARS-CoV-2 epidemic curve in Johannesburg's Region F in South Africa between 22 November 2020 and 01 August 2021.

Figure 2 depicts the proportion of SARS-CoV-2 seropositive pregnant women per epidemiologic week at the two study sites, plotted against the backdrop of the SARS-CoV-2 epidemic curve in Johannesburg's Region F. Peaks and troughs are evident at similar time points for both clinics. However, a divergence in the seroprevalence was noted in the last week of the study, possibly due to the small number of participants enrolled at that time. Seroprevalence was highest in April and May 2021 (65.9% and 64.4%, respectively) (Table 4), which coincided with the start of the third wave of infections in South Africa, which was characterized by the highly transmissible Delta variant.

**Table 4:** Factors associated with SARS-CoV-2 antibody detection using two methods of detection in Johannesburg, South Africa, March-June 2021: (i) EITHER Wantai or Roche assay detection; and (ii) BOTH Wantai and Roche assay detection.

	Antibodies detected on EITHER Wantai or Roche Assay		Antibodies detected on BOTH Wantai and Roche Assays	
Characteristics	SARS-CoV-2 antibodies detected n/N (%)	Unadjusted prevalence ratios^b^ (95% CI)	SARS-CoV-2 antibody detected n/N (%)	Unadjusted prevalence ratios^b^ (95% CI)
**Overall**	320/500 (64.0)	-	270/500 (54.0)	-
**Month of enrollment**				
March 2021	68/110 (61.8)	Reference	61/110 (55.5)	Reference
April 2021	112/170 (65.9)	1.07 (0.79-1.44)	92/170 (54.1)	0.98 (0.71-1.34)
May 2021	114/177 (64.4)	1.04 (0.77-1.41)	92/177 (52.0)	0.94 (0.68-1.29)
June 2021	26/43 (60.5)	0.98 (0.62-1.54)	25/43 (58.1)	1.05 (0.66-1.67)
**Median age (IQR) in years**	27.46 (23.42-31.63)	0.99 (0.98-1.01)	27.69 (23.35-31.97)	0.99 (0.98-1.02)
**Age group**				
15-19	17/27 (63.0)	0.99 (0.59-1.66)	16/27 (59.3)	1.11 (0.65-1.90)
20-24	94/148 (63.5)	Reference	79/148 (53.4)	Reference
25-29	101/150 (67.3)	1.06 (0.80-1.40)	81/150 (54.0)	1.01 (0.74-1.38)
30-34	65/106 (61.3)	0.97 (0.70-1.32)	54/106 (50.9)	0.95 (0.66-1.35)
35-39	38/58 (65.5)	1.03 (0.71-1.50)	35/58 (60.3)	1.13 (0.76-1.68)
≥40	5/11 (45.5)	0.72 (0.29-1.76)^a^	5/11 (45.5)	0.85 (0.34-2.10)
**Clinic/Site**				
**Esselen Street Clinic**	149/250 (59.6)	Reference	126/250 (50.4)	Reference
**Shandukani Midwife Obstetric Unit**	171/250 (68.4)	1.15 (0.92-1.43)^a^	144/250 (57.6)	1.14 (0.90-1.45)
**Nationality**				
South African	157/253 (62.1)	Reference	134/253 (52.9)	Reference
Zimbabwean	152/231 (65.8)	1.06 (0.85-1.33)	126/231 (54.6)	1.03 (0.81-1.31)
Other	11/16 (68.8)	1.11 (0.60-2.04)	10/16 (62.5)	1.18 (0.62-2.24)
**Parity**				
0	118/182 (64.8)	Reference	104/182 (57.2)	Reference
1	113/176 (64.2)	0.99 (0.63-1.50)	92/176 (52.3)	0.91 (0.69-1.21)
2-4	87/135 (64.4)	0.99 (0.62-1.57)	73/135 (54.1)	0.95 (0.70-1.27)
≥ 5	1/1 (100)	1.54 (0.22-11.04)	1/1 (100)	1.75 (0.24-12.54)
**Gravidity**				
1	99/156 (63.5)	Reference	85/156 (54.5)	Reference
2-4	208/320 (65.0)	1.02 (0.81-1.30)	175/320 (54.7)	1.00 (0.77-1.30)
≥ 5	12/18 (66.7)	1.05 (0.58-1.91)	10/18 (55.6)	1.02 (0.53-1.96)
**Median gestational age of fetus (IQR) (weeks)**				
	27 (21-34)	0.99 (0.98-1.01)	27 (21-34)	0.99 (0.98-1.01)
**Trimester of pregnancy**				
First trimester	7/12 (58.3)	0.91 (0.43-1.95)	5/12 (41.67)	0.77 (0.32-1.89)
Second trimester	147/223 (65.9)	1.03 (0.83-1.29)	126/223 (56.5)	1.05 (0.83-1.34)
Third trimester	165/259 (63.7)	Reference	139/259 (53.7)	Reference
**HIV status**				
Negative	238/360 (66.1)	Reference	204/360 (56.7)	Reference
Positive	76/131 (58.0)	0.88 (0.68-1.14)	61/131 (46.6)	0.82 (0.62-1.09)^a^
**HIV viral load (copies/ml)**				
<100	36/69 (52.2)	Reference	32/69 (46.4)	Reference
≥100	7/12 (58.3)	1.11 (0.50-2.51)	5/12 (41.7)	0.90 (0.35-2.31)
**Clusters of differentiation 4+ T cells (count/ml)**				
≥350	24/39 (61.5)	Reference	21/39 (53.85)	Reference
<350	7/18 (38.9)	0.63 (0.27-1.47)	4/18 (22.2)	0.41 (0.14-1.20)^a^
**Syphilis**				
Negative	285/444 (64.2)	Reference	241/444 (54.3)	Reference
Positive	10/17 (58.8)	0.92 (0.49-1.72)	9/17 (52.9)	0.98 (0.50-1.90)
**Symptoms suggestive of any previous COVID infection (since March 2020)**				
Yes	11/19 (57.9)	0.90 (0.49-1.64)	10/19 (54.1)	0.98 (0.52-1.83)
No	309/481 (64.2)	Reference	260/481 (52.6)	Reference
**Prior positive COVID test**				
Yes	6/6 (100.0)	1.57 (0.70-3.53)	6/6 (100.0)	1.87 (0.83-4.20)^a^
No	314/494 (63.6)	Reference	264/494 (53.4)	Reference
**Willing to take COVID-19 vaccine when available**				
Yes	182/296 (61.5)	Reference	157/296 (55.5)	Reference
No/Unsure	136/202 (67.3)	1.09 (0.88-1.37)	112/202 (55.5)	1.04 (0.82-1.33)

a *P*-values <0.25 on univariate regression

b Multivariable logistic regression results not shown. None of the candidate variables from the univariate regression became significant predictors of the outcome when assessed together in a multivariable model. CI, confidence interval; IQR, interquartile range

### Factors associated with seropositivity

In the univariate analyses, we observed no clinical or demographic factors that were significantly associated with SARS-CoV-2 detection when using both the primary measure of seropositivity (antibody detection on either the Wantai or the Roche assay) and the secondary measure (antibody detection on both the Wantai and the Roche assay) (Table 4).

Two candidate variables were eligible for inclusion in the multivariable model: study site and age categories. However, neither of these weakly associated variables became significant predictors of the outcome when assessed together in a multivariable model. In a separate multivariable model restricted to WLHIV, based on the literature, we also included the indicators for HIV viral load elevation and CD4 suppression as variables of clinical importance. Again, none of the variables assessed became significant predictors of the outcome when taken together in the multivariable model. Results from these multivariable analyses are not reported.

Only 3.4% (11/320) of the women who were seropositive reported ever having symptoms suggestive of COVID-19 infection.

### Willingness to vaccinate

Only 296 (59.4%) women reported being willing to be vaccinated should vaccines become available, whereas the rest (40.6%) reported being unsure or unwilling to be vaccinated. More women (174/250, 69.6%) at the Esselen Street Clinic were willing to receive a COVID-19 vaccine than at Shandukani Midwife Obstetric Unit (122/250, 48.8%, *P* <0.001) (Supplementary Table 1).

## Discussion

Between March and June 2021, we enrolled 500 pregnant women attending two high-volume antenatal clinics in a densely populated inner-city area of Johannesburg, with an antenatal HIV seroprevalence of about 30%. A high proportion, 64.0%, of pregnant women had evidence of a previous SARS-CoV-2 infection. Overall, 96.6% of the seropositive women did not recall having any symptoms of SARS-CoV-2 infection between March 2020 and the time of enrollment.

This serosurvey was conducted between South Africa's second and third waves. Given that IgG antibodies most commonly become detectable at 1-3 weeks after infection (Sethuraman *et al.*, 2020), it is highly likely that the seroprevalence detected in this study is due to the cumulative infections that occurred in the first and second waves, with a small proportion likely attributable to newer infections experienced at the start of the third wave. Previous seroprevalence surveys in pregnant women in South Africa have reported rates of between 30.8% in Gauteng and 38% in the Western Cape after the first wave of infections (George *et al.*, 2021; Hsiao *et al.*, 2020). A study among blood donors in South Africa, conducted after the peak of the second wave, reported seroprevalences ranging from 32-63% among four provinces (Sykes *et al.*, 2021). A cross-sectional study conducted in three communities in South Africa, during and after the second wave of infection, reported an increase in seropositivity from 26.9% in December 2020 to 47.2% in April 2021 (Wolter *et al.*, 2022). These findings across all studies do not take into consideration the potential for waning antibody levels or seroreversion over time (Meyer, 2021; Peluso *et al.*, 2021). This sequential increase in the seroprevalence with subsequent waves of infection and high seroprevalence gives credence to the fact that South Africa has experienced a more pervasive epidemic than estimated when using laboratory-based RT-PCR or antigen-diagnosed cases, which are mostly symptomatic (Hsiao *et al.*, 2020), resulting in a substantial underestimation of the proportion of the population previously infected. Repeated seroprevalence studies are likely a more accurate indicator of infection prevalence than RT-PCR/antigen-based case identification. Serial seroprevalence studies of pregnant women are easily implementable and could be built into routine blood testing during antenatal clinic visits because pregnant women have regularly scheduled clinical appointments. These studies among pregnant women as a sentinel population will allow for timely, efficient, and low-cost determination of the prevalence of immunity, changes in immunity over time, durability of immune protection, vaccine uptake and vaccine penetrance over time.

Among WLHIV, Hsaio *et al.* (2020) reported that 42% seroprevalence after the peak of the first wave of the epidemic among women attending antenatal clinics in Cape Town Metropolitan (Metro) subdistricts. As expected, we found a higher seroprevalence of 58.0% among WLHIV in Johannesburg after two waves of infection. WLHIV with a low CD4 cell count (<350 count/ml) were less likely to be seropositive on the Roche Elecsys assay (PRR = 0.39; 95% CI: 0.16-0.98) than women with higher CD4 counts; although, this analysis was restricted to only 57 of the 131 WLHIV, in whom CD4 cell counts were available. In addition to the four seropositive cases detected on both assays, the Wantai assay detected an additional three seropositive cases among WLHIV with a low CD4 cell count, most likely due to the Wantai assay measuring anti-S antibodies. Individuals living with HIV who are immunocompromised have been shown to be less likely to develop a SARS-CoV-2 antibody response (Meiring *et al.*, 2022; Wolter *et al.*, 2022) as well as have lower IgG concentrations and pseudovirus neutralizing antibody titers than individuals without HIV (Spinelli *et al.*, 2021; Wolter *et al.*, 2022). In addition, anti-N antibodies have been shown to wane faster than anti-S antibodies after infection (Choudhary *et al.*, 2021; Lumley *et al.*, 2021). Therefore, WLHIV who are immunocompromised were less likely to be seropositive and were more likely to test positive on the anti-S assay than the anti-N assay. A combination of the Roche Elecsys and Wantai assays in this study produced a higher yield of SARS-CoV-2 seroprevalence. Nevertheless, it is possible that serology may underestimate previous infection among WLHIV, especially those with advanced immunosuppression.

Studies evaluating clinical course and pregnancy outcomes in women infected with SARS-CoV-2 during pregnancy showed high rates of intensive care unit admission, Cesarean section delivery, higher maternal mortality, and preterm delivery (Budhram *et al.*, 2021; Carrasco *et al.*, 2021; Della Gatta *et al.*, 2020). In our study, we were unable to ascertain when SARS-CoV-2 infection occurred, except in those who reported a positive SARS-CoV-2 antigen test. It is reassuring that in our study population, many women have some expected level of immunity while they are pregnant because of a previous natural infection. Previous SARS-CoV-2 infection is expected to have some level of protection against repeat SARS-CoV-2 infection (although, this may not hold for newer variants of concern). Similarly, one COVID-19 vaccine with a previous infection results in robust immunity that is comparable to two vaccines (Bertollini *et al.*, 2021; Keeton *et al.*, 2021; Shrestha *et al*., 2022).

During the study period, only 56% of pregnant women reported willingness to be vaccinated against COVID-19. Apart from the observed difference between clinic sites, we found no other demographic or clinical factors associated with the willingness to be vaccinated. This study did not investigate reasons for vaccine hesitancy. However, other studies of SARS-CoV-2 vaccine hesitancy, conducted before vaccine availability to the public in South Africa, report variable levels of survey respondents indicating a willingness to accept COVID-19 vaccination, ranging from 52-82% (Cooper *et al.*, 2021; Skjefte *et al.*, 2021). Factors potentially associated with willingness to be vaccinated included older age, urbanicity, geographic location, and female sex (Cooper *et al.*, 2021; Skjefte *et al.*, 2021). A common theme in these studies, was concern about COVID-19 vaccine safety and effectiveness, highlighting the need for communication and education strategies specific for populations of interest to encourage vaccination (Cooper *et al.*, 2021). The willingness to vaccinate among women in this study may have also been influenced by the lack of guidelines for vaccinations in pregnant women at the time. At the end of August 2021, after the completion of our study, the South African Department of Health released a circular recommending all pregnant and lactating women be vaccinated with either of the available vaccines.

### Limitations

This study had several limitations. We experienced a high refusal rate among women attending routine antenatal visits, resulting in a possible selection bias in women who enrolled, although very few enrolled women reported symptoms or previous positive SARS-CoV-2 tests. The study was conducted at two antenatal facilities within a limited geographical area, thus potentially limiting the generalizability to other urban environments. The cross-sectional design of the survey leads to uncertainty of the timing of previous SARS-CoV-2 infections, potential recall bias of symptoms experienced up to 1 year before study enrollment, and the potential for an underestimation of SARS-CoV-2 infection due to waning antibody levels among women who were infected in the first wave. It is possible that the proportion of women experiencing symptomatic SARS-CoV-2 infection was underestimated, though another study conducted in South Africa at a similar time also reported low proportions (3.4%) of individuals who were seropositive and symptomatic (Wolter *et al.*, 2022). This study only detected binding antibodies; we did not determine whether these were neutralizing antibodies with the ability to neutralize the SARS-CoV-2 virus. Lastly, we did not collect information on comorbidities and risk factors for SARS-CoV-2 disease, such as diabetes, obesity, and socioeconomic status, which have been found to be associated with SARS-CoV-2 seropositivity in other studies in South Africa (George *et al.*, 2021; Wolter *et al.*, 2022). Given that almost 70% of the population of Johannesburg is overweight or obese (Nordisk, 2016) and many have a low socioeconomic status (Rees *et al.*, 2017), we postulate that we may have seen similar associations between these risk factors and seropositivity.

## Conclusion

This easily accessible population of pregnant women attending routine antenatal care visits had a high SARS-CoV-2 seroprevalence, with most women experiencing asymptomatic disease. WLHIV and those with immune suppression were less likely to have anti-N antibodies and were therefore less likely to have anti-N antibodies detected on the Roche assay. Seroprevalence surveys could present a feasible, low-cost method of monitoring the course of the pandemic, vaccine uptake, and vaccine hesitancy in a population and should ideally be conducted after each wave of infection.

## Declaration of competing interest

CC has received grant support from Sanofi Pasteur and Advanced Vaccine Initiative and payment of travel costs from Parexel. NW and AvG have received grant support from Sanofi Pasteur and the Bill and Melinda Gates Foundation.

## Appendix Supplementary materials

Image, application 1
