# Supplementary material for: High prevalence of SARS-CoV-2 antibodies in pregnant women after the second wave of infections in the inner-city of Johannesburg, Gauteng Province, South Africa
Source: Int J Infect Dis. 2022 Dec;125:241–9. doi: 10.1016/j.ijid.2022.10.036 (PMC9637015; doi:10.1016/j.ijid.2022.10.036)
Supplement: Supplementary file 1 [file mmc1.docx]

**Supplementary Table 1**: Factors associated with willingness to receive a COVID-19 vaccine, Johannesburg, South Africa, March – June 2021

| Characteristics | N | Willing,  n (%N) | Unwilling or unsure,  n (%N) | Unadjusted Prevalence Ratios^1^  (95% CI) |
| --- | --- | --- | --- | --- |
| Total | 498 | 296 (59.4) | 202 (40.6) |  |
| Age group  15-19  20-24  25-29  30-34  35-39  ≥40 | 27  147  150  105  58  11 | 15 (55.6)  76 (51.7)  92 (61.3)  66 (62.9)  40 (69.0)  7 (63.6) | 12 (44.4)  71 (48.3)  58 (38.7)  39 (37.1)  18 (31.0)  4 (36.4) | 1.07 (0.62 – 1.87)  Reference  1.19 (0.88 – 1.61)  1.22 (0.87 – 1.69)  1.33 (0.91 – 1.96)  1.23 (0.57 – 2.67) |
| Clinic/Site  Esselen Street Clinic  Shandukani MOU | 248  250 | 174 (70.2)  122 (48.8) | 74 (29.8)  128 (51.2) | Reference  **0.70 (0.55 – 0.88)** |
| Nationality  South African  Zimbabwean  Other | 252  230  16 | 143 (56.7)  144 (62.6)  9 (56.3) | 109 (43.3)  86 (37.4)  7 (43.7) | Reference  1.10 (0.88 – 1.39)  0.99 (0.51 – 1.94) |
| Parity  0  1  2-4  ≥ 5 | 182  175  134  1 | 96 (52.8)  106 (60.6)  88 (65.7)  1 (100) | 86 (42.2)  69 (39.4)  46 (34.3)  0 (0) | Reference  1.15 (0.87 – 1.51)  1.25 (0.93 – 1.66)  1.90 (0.26 – 13.60) |
| Gravidity  1  2-4  ≥ 5 | 156  318  18 | 83 (53.2)  198 (62.3)  10 (55.6) | 73 (46.8)  120 (37.7)  8 (44.4) | Reference  1.17 (0.91 – 1.51)  1.04 (0.54 – 2.01) |
| Trimester of pregnancy  1^st^ Trimester  2^nd^ Trimester  3^rd^ trimester | 12  223  259 | 9 (75.0)  133 (59.6)  149 (58.0) | 3 (25.0)  90 (40.4)  108 (42.0) | 1.26 (0.64 – 2.47)  Reference  0.97 (0.77 – 1.23) |
| HIV status  Known  Unknown/missing | 489  9 | 292 (59.7)  4 (44.4) | 197 (40.3)  5 (55.6) | Reference  0.74 (0.28 – 2.00) |
| HIV status  Positive  Negative | 131  358 | 86 (65.6)  206 (57.5) | 45 (34.4)  152 (42.5) | 1.14 (0.89 – 1.47)  Reference |
| HIV viral load (copies/ml)  <100  ≥100 | 69  12 | 46 (66.7)  8 (66.7) | 23 (33.3)  4 (33.3) | Reference  1.00 (0.47 – 2.12) |
| CD4+ T cells (count/ml)  ≥350  <350 | 39  18 | 28 (71.8)  16 (88.9) | 11 (28.2)  2 (11.1) | Reference  1.23 (0.67 – 2.29) |
| Prior positive SARS-CoV-2 PCR test  Yes  No | 6  492 | 3 (50.0)  293 (59.5) | 3 (50.0)  199 (40.5) | 0.84 (0.27 – 2.62)  Reference |
| Symptoms suggestive of any previous COVID infection (since Mar 2020)  Yes  No | 19  479 | 13 (68.4)  283 (59.1) | 6 (31.6)  196 (40.9) | 1.15 (0.66 – 2.02)  Reference |
| SARS-CoV-2 Antibodies detected on EITHER Wantai or Roche Assay  Positive  Negative | 318  180 | 182 (57.2)  114 (63.3) | 136 (42.8)  66 (36.7) | 0.90 (0.72 – 1.14)  Reference |

^1^ Only one variable in univariate regression had a p-value<0.25, therefore multivariable regression was not conducted.
